# Apneic laryngeal oxygenation during elective fiberoptic intubation – a technical simulation

**DOI:** 10.1186/s12871-020-01216-2

**Published:** 2020-12-09

**Authors:** Daniel C. Schroeder, Wolfgang A. Wetsch, Simon-Richard Finke, Fabian Dusse, Bernd W. Böttiger, Holger Herff

**Affiliations:** University of Cologne, Faculty of Medicine and University Hospital of Cologne, Department of Anaesthesiology and Intensive Care Medicine, Kerpener Strasse 62, 50937 Cologne, Germany

**Keywords:** Apneic oxygenation, Oropharyngeal oxygenation device, Oxygen desaturation, Simulation

## Abstract

**Background:**

Sedation during elective fiberoptic intubation for difficult airway can cause respiratory depression, apnea and periods of desaturation. During apneic episodes, hypoxemia can be prevented by insufflation of oxygen in the deep laryngeal space. The aim of this study was to evaluate an oropharyngeal oxygenation device (OOD) designed for deep laryngeal insufflation during fiberoptic intubation.

**Methods:**

The OOD is split in the front to form a path for the bronchoscope. An external lumen delivers oxygen in the deep laryngeal space. In this experimental study, air application (as control group), oxygen application via nasal prongs, oxygen application via the OOD, and oxygen application via the working channel of a bronchoscope were compared in a technical simulation. In a preoxygenated test lung of a manikin, decrease of the oxygen saturation was measured over 20 min for each method.

**Results:**

Oxygen saturation in the test lung dropped from 97 ± 1% (baseline in all groups) to 58 ± 3% in the control-group (*p* < 0.001 compared to all other groups) and to 78 ± 1% in the nasal prong group (*p* < 0.001 compared to all other groups). Oxygen saturation remained at 95 ± 2% in both the OOD group and the bronchoscopy group (*p* = 0.451 between those two groups).

**Conclusion:**

Simulating apneic laryngeal oxygenation in a preoxygenated manikin, both oxygen insufflation via the OOD and the bronchoscope kept oxygen saturation in the test lung at 95% over 20 min. Both methods significantly were more effective than oxygen insufflation via nasal prongs.

## Background

Elective fiberoptic intubation is usually performed in patient with difficult airways and with a serious risk for development of hypoxemia [1]. Despite comprehensive preoxygenation, which is mandatory prior to initiation of any airway management, peripheral oxygenation of less than 90% regularly occurs [2–4]. In fact, patients undergoing fiberoptic intubation are often sedated, which may result in loss of protective oral reflexes and hence a partial or even complete obstruction of the upper airways, or a drug induced hypoventilation.

To prevent hypoxemia during fiberoptic intubation, there is a variety of measures, including dedicated face masks for oxygen insufflation, non-invasive ventilation (NIV) and high-flow oxygenation (HFO) by nasal cannulas [5–8]. Although severe hypoxemic events can often be prevented by these measures, episodes of hypoxemia and peripheral oxygen desaturation frequently occurred in several studies [6–8]. Presumably, oxygen applied through face- and NIV-masks or HFO-cannulas is not able to pass through the partially obstructed upper airway of sedated patients. Additionally, the majority of face masks as well as HFO-cannulas are at risk of dislocating during the procedure. Furthermore, they may require an airtight seal, which is difficult to be achieved in toothless, overweight or bearded patients [5]. Moreover, a full airtight seal cannot be achieved when attempting fiberoptic intubation, which drastically reduces the effectiveness and safety of these devices.

**Fig. 1:**
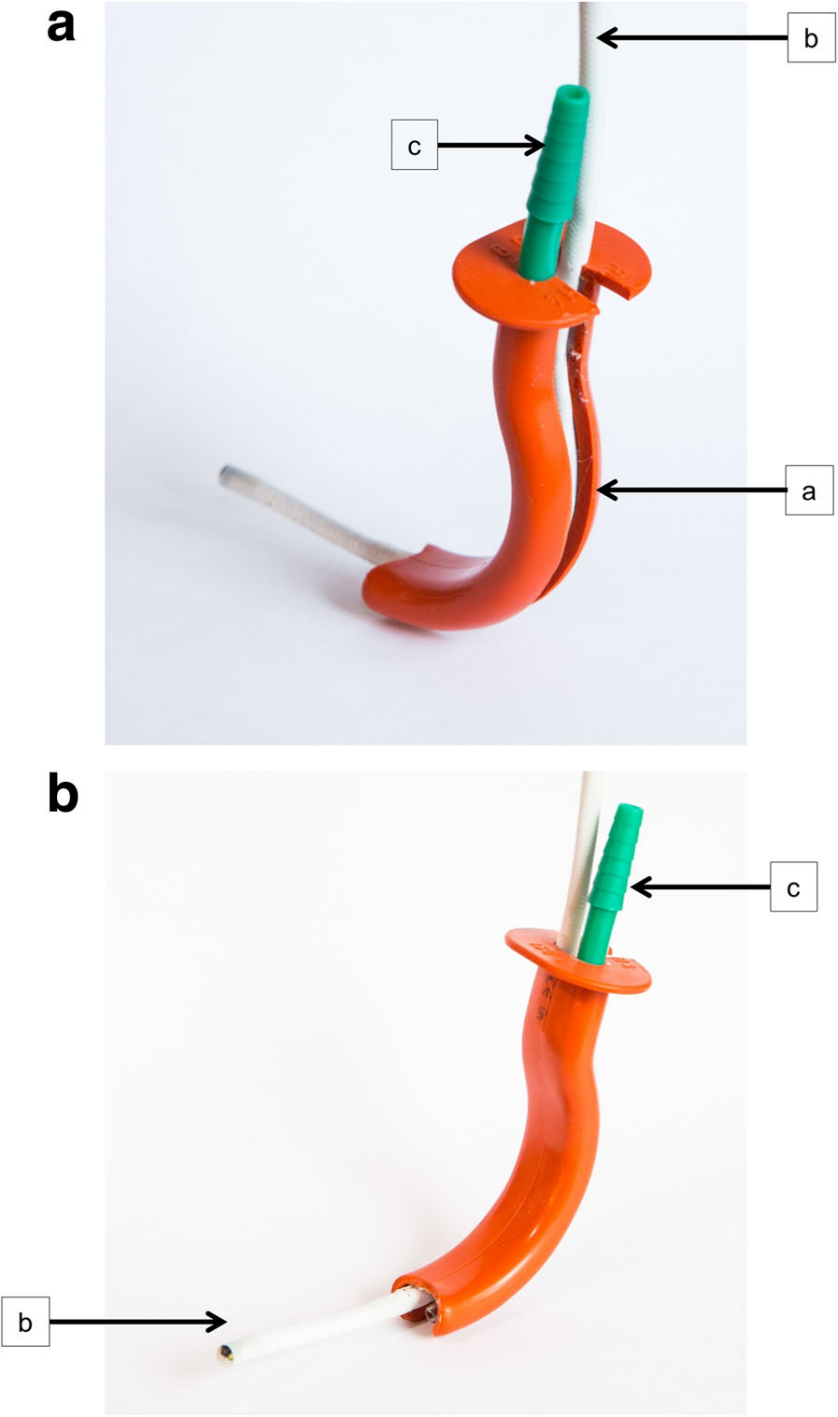
Prototype of an oropharyngeal oxygenation device (OOD) from the back (**A**) and the front side (**B**). The OOD is split to form a path (a) for the bronchoscope (b), with an external lumen to provide oxygen insufflation into the deep laryngeal space (c)

There is a scientific body of evidence that hypoxemia can be prevented by deep insufflation of oxygen in the laryngeal space and trachea during apneic episodes [9–12]. Mitterlechner et al. reported that oxygen saturation in a test-lung of a manikin remains significantly higher when using a specific laryngoscope blade designed for laryngeal oxygen insufflation, compared to nasal oxygen insufflation [9]. Additionally, O’Loughlin et al. showed that low-flow tracheal apneic oxygenation during microlaryngoscopy under general anesthesia allowed a successful conduction of surgical procedures and also impeded the increase of carbon-dioxide levels [11]. However, while oxygenation concepts approaching deeper anatomical airway structures are scientifically proven for unexpected difficult airways [13], evidence for apneic laryngeal oxygenation during elective fiberoptic intubation is scarce. In this study, we evaluated a special oropharyngeal oxygenation device (OOD), allowing a continuous laryngeal oxygen insufflation during and parallel with bronchoscopy. In detail, this device consists of an external oxygen line inserted into a special “split” oropharyngeal tube. The split tube construction allows more space for the bronchoscope. Combining these, both continuous oxygen insufflation into the laryngeal space and bronchoscopy can be conducted simultaneously (Fig. 1).

Aim of this study was to scientifically evaluate the effectiveness of an OOD in a technical simulation for the first time. To avoid possible harm for patients, we chose a technical simulation with an airway manikin. Prior to the beginning, the test lungs of the manikin were filled with pure oxygen (“preoxygenated”). Subsequently, four different strategies of oxygen insufflation were evaluated: The first strategy was a control group allowing ambient air to enter the manikins airway, the second strategy was oxygen application via nasal prongs, the third strategy was oxygen insufflation via a special OOD, and the fourth strategy was oxygen insufflation via the working channel of a bronchoscope into the deep laryngeal space. Decrease in oxygen saturation in the test lung was measured for 20 min. We hypothesized that there would be no difference in oxygen fraction decrease between the groups.

## Methods

### Ethics approval

This study is a completely technical simulation with no participants. Thus, no ethical approval was required.

### Experimental setup

The trachea of an anatomically correctly shaped male manikin (Laerdal®-Airwaymanagement-Trainer, Laerdal Medical GmbH, Puchheim, Germany), was attached to a test lung with a volume of 2.5 L, simulating the functional residual capacity of an adult man. Caliber of nostrils and degree of mouth opening were determined by the dummy and could not be altered. Before each experiment, the test lung was preoxygenated with pure oxygen to an oxygen saturation of 97% using an anaesthesia machine (Draeger Primus®, Drägerwerk AG & Co. KGaA, Lübeck, Germany). Subsequently, the test lung was connected to a gas analyzer sampling system with a suction rate of 200 mL/min (Draeger Primus®, Drägerwerk AG & Co. KGaA, Lübeck, Germany), a rate that is comparable to the oxygen consumption of an adult during apnea [14]. The sample was taken from the base of the test lung. The oxygen sensor itself is based on an electrochemical principle (Galvanic cell) containing sodium hydroxide and lead.

### Experimental procedure

Before initiation of each experiment, interventional procedures were randomly assigned to an experimental group. *Control group:* no intervention, ambient air enters the manikin’s airway. *Nasal prong group:* oxygen was insufflated through a standard one-line nasal prong. As recommended, application of 4 L/min oxygen through nasal prongs was not exceeded. To precisely compare the results, 4 L/min was also chosen in the other groups. *Oropharyngeal oxygenation device group (OOD group):* oxygen was insufflated via the external line of the oropharyngeal oxygenation device (Ruesch, Rommelshausen, Germany). *Bronchoscopy group:* oxygen was insufflated through the working channel of a bronchoscope (AMBU® aScope Broncho Regular, AMBU A/S, Ballerup, Denmark) placed in the oropharyngeal oxygenation device, while its external line was not used for oxygen insufflation.

Six experiments per group were conducted to achieve sufficient statistical power. Primary objective was the decrease in oxygen saturation within a period of 20 min after preoxygenation of the test-lung. Measurements of oxygen saturation were performed by an observer blinded to the method of oxygen delivery.

### Statistical analysis

Data are reported as mean plus or minus standard deviation. After checking for normality of distribution using Kolmogorov-Smirnov test, a one-way analysis of variance (ANOVA) for repeated measurements was performed to determine overall statistical significance between groups, followed by post hoc Tukey test for pair wise multiple comparisons (Sigmaplot 14; Systat, San Jose, CA); *p* < 0.05 was considered significant.

## Results

**Fig. 2:**
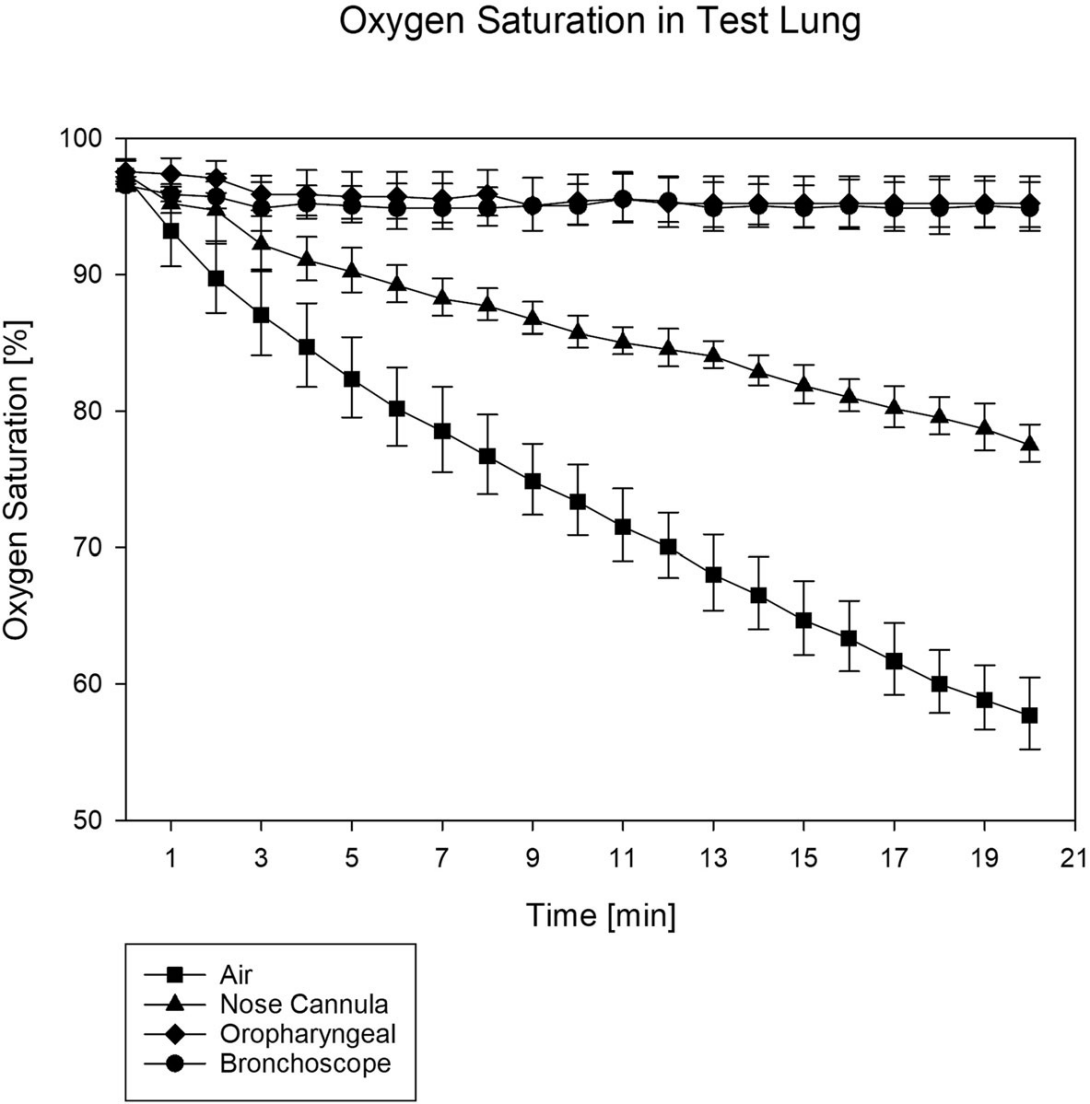
Oxygen saturation in the test lung vs. time; *p* = 0.451 between the OOD group and the bronchoscopy group; *p* < 0.001 between all other groups

A one-way ANOVA for repeated measurements detected statistical significance between all groups (*p* < 0.001). During the 20-min observation period, oxygen saturation in the test lung dropped from 97 ± 1% at baseline in all groups to 58 ± 3% (95% CI 55–61) in the control group (*p* < 0.001 compared to all other groups) and to 78 ± 1% (95% CI 76–79) in the nasal prong group (*p* < 0.001 compared to all other groups). Oxygen saturation remained at 95 ± 2% (95% CI 93–97) in the oropharyngeal oxygenation device group and at 95 ± 2% (95% CI 93–97) in the bronchoscopy group (*p* = 0.451 between these two groups, Fig. 2).

## Discussion

In this technical simulation, insufflating oxygen in the deep laryngeal space via an OOD was more effective than nasal oxygen insufflation, and comparable effective to oxygen insufflation via the working channel of a bronchoscope.

Performing careful preoxygenation has proven to reduce harmful deoxygenation during elective fiberoptic intubation [15]. Applying additional apneic laryngeal oxygen may reduce episodes of hypoxemia in spontaneously breathing patients that suffer respiratory depression due to sedating agents for fiberoptic intubation. Thus, laryngeal oxygen insufflation via an OOD may provide a simple and rapid additional oxygenation strategy in planned fiberoptic intubation. The 20 min of apneic oxygenation chosen in this study may seem long for a normal standard fiberoptic intubation; e.g. Lee et al. reported in a recent study of an average of 72 s to achieve a successful fibreoptic intubation [16]. However, Popat et al. reported in the Fourth National Audit Project of the Royal College of Anaesthetists and the Difficult Airway Society that severe problems during fiberoptic intubation occur regularly. In many of these cases, experienced anesthetists even failed to intubate successfully [17]. In these cases, 20 min may be realistic for prolonged fiberoptic intubation attempts by different anesthetists and the time needed to return to spontaneous ventilation after stopping sedation, if fiberoptic intubation finally fails. In this model, oxygen insufflation via the OOD was comparably effective to insufflation via the bronchoscope. However, oxygen insufflation regularly blocks the working channel of the bronchoscope, which is regularly required for suctioning saturation from the upper airway, and to maintain a clear bronchoscopic view, which is the prerequisite for successful fiberoptic intubation. In contrast, apneic laryngeal administration of oxygen via the OOD provides both adequate flow of oxygen and availability of the bronchoscope’s working channel for suctioning.

Not surprisingly, the decrease in oxygen saturation in the test lung was delayed by nasal oxygen insufflation compared to the control group using air, which is in accordance with previous studies [9]. Patel et al. reported about transnasal humidified rapid-insufflation ventilatory exchange (THRIVE) applying oxygen at high flows which may be even more effective [18]. However, these devices apply a completely different principle: THRIVE creates continuous positive airway pressure (CPAP) inside the oral cavity, thus preventing an obstruction at the level of distal oral cavity. The flow of 4 L/min that we choose in this experiment cannot relieve the obstruction at the level of velopharynx. Further, actual anesthesia guidelines postulate to apply “a bulk flow of oxygen” via nasal cannula e.g. for the management of difficult airways in obstetric anesthesia [19]. Although nasal high flow oxygenation is promising [20, 21], deep nasal cannulas may be accompanied by further adverse effects including the risk of nasal bleeding, that could make airway management even more difficult [22]. Further, high flow devices as THRIVE need special technical applications that may not be available in every anesthesia workplace, whereas 4 L/min of oxygen should be applicable nearly everywhere. In addition to that, in patients suffering from an upper airway obstruction, a bronchoscope further restricts the diameter of the trachea, which may result in lung-distension or tracheal injuries.

Our results indicate that oxygen insufflation via OOD or bronchoscope is more effective than nasal oxygen insufflation. Due to multiple angulations, upper airway anatomy can cause turbulent flow, which may result in mixing of oxygen applied via nasal prongs with nitrogen entering through the manikin’s mouth [9]. In contrast, during deep laryngeal insufflation, oxygen emits retrogradely through the manikin’s mouth, which obviously is more effective for maintenance of continuous denitrogenization of the airway [22]. Advantageously, the splitted OOD keeps the upper airway passable, which may also support denitrogenization.

The artificial nature of a simulation scenario is a limitation by itself; e.g., we were not able to simulate carbon dioxide production. Thus, after 20 min of apneic oxygenation, hypercarbia and acidemia would have developed in a living organism. However, as long as the patient remains hyperoxic, this can be tolerated for comparable times, as reported previously [23]. We further did not simulate humidification of gases, and last suctioned out all gases from the test lung using the oxymeter. During apneic oxygenation, nitrogen that enters the lungs does not dissolve in the completely nitrogen-saturated blood and thus accumulates in the lungs. In contrast, by suctioning nitrogen out of the test lungs, our model may have underestimated the speed of the decrease of oxygen saturation in the test lung, compared to a living organism. Creating a more realistic scenario would have required an animal experiment, which has to be done in the future. However, human studies would have needed large numbers of participants, since deoxygenation due to hypopnea does not occur in a majority of cases. Thus, we assumed our model to be sufficiently realistic and the best available method to test our hypothesis in a human airway model. A further limitation of the device itself is that it cannot be inserted when mouth opening is limited - one of the most common indications to perform awake fibreoptical intubation.

Another point to be considered is that an oxygen flow of 4 L/min was used, which is recommended for nasal prongs. Possibly, a higher oxygen flow via nasal prongs would have led to higher oxygenation of the test-lung and thereby attenuated the differences between the groups. However, to scientifically compare the measures in an appropriate manner, we chose an oxygen flow of 4 L/min in each group. We deliberately choose this oxygen flow of 4 L/min per minute since that should be available in every operation theatre; we further used this flow in all devices (apart from the control group) to isolate the influence of the devices on oxygen concentration in the lungs in this proof of principle study.

We used an in-house manufactured oropharyngeal oxygenation device. It has to be mentioned that a similar device is available on the market (MADgic AirwayTM, Teleflex Medical Europe, Dublin, Ireland). The authors want to emphasize that there is no relation or commercial activity to the manufacturer influencing the current study that was intended to gain new insights in the concept of apneic laryngeal oxygenation. Further, adequate oxygenation can be achieved using other simple or more highly sophisticated devices e.g. laryngeal insufflation via a suction catheter of 10 to 15 cm length, or a pediatric endotracheal tube that can be easily placed in the hypopharynx. There are also distinct devices such as the Ovassapian or William’s intubating airway. In our opinion, the key factor is the placement of the oxygen supply tube in the lower hypopharynx to maintain adequate oxygenation in this model.

Having established themselves as kind of standard for oxygenation in pediatric patients, nasal HFO has gained popularity in adult patients. As HFO may also be able to deliver oxygen in the hypopharynx, future research should also include the use of nasal high-flow oxygen as comparator.

In conclusion, both oxygen insufflation via the OOD and the bronchoscope kept oxygen saturation in the test lung at 95% over 20 min. The results of this study suggest that oxygenation via OOD or bronchoscope can prevent desaturation better than nasal oxygen at 4 l/min. Under clinical conditions, an OOD may be considered as alternative to prevent hypoxemia in difficult airway scenarios.

## Data Availability

All data generated used or analyzed during this study are included in the published article.

## Abbreviations

ANOVA: Analysis of variances
CPAP: Continuous positive airway pressure
HFO: High-flow oxygenation
NIV: Non-invasive ventilation
OOD: Oropharyngeal oxygenation device
THRIVE: Transnasal humidified rapid-insufflation ventilatory exchange
